# Supplementary figures and images for: Impact of Preoperative Visceral Fat Area Measured by Bioelectrical Impedance Analysis on Clinical and Oncologic Outcomes of Colorectal Cancer
Source: Nutrients. 2022 Sep 24;14(19):3971. doi: 10.3390/nu14193971 (PMC9572030; doi:10.3390/nu14193971)

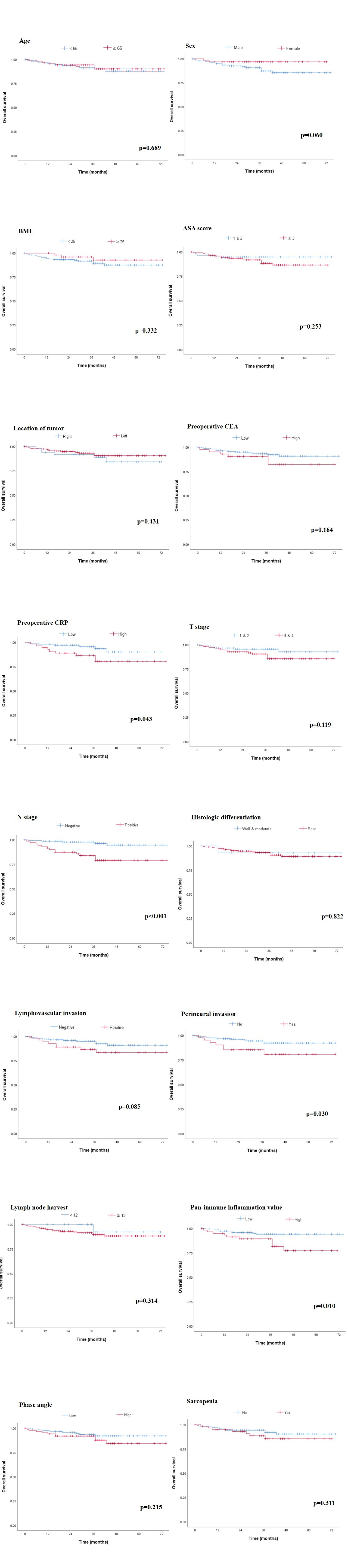

Supplement: Supplementary file 1 [file nutrients-14-03971-s001.zip › Supplementary-Figure S1.tiff]

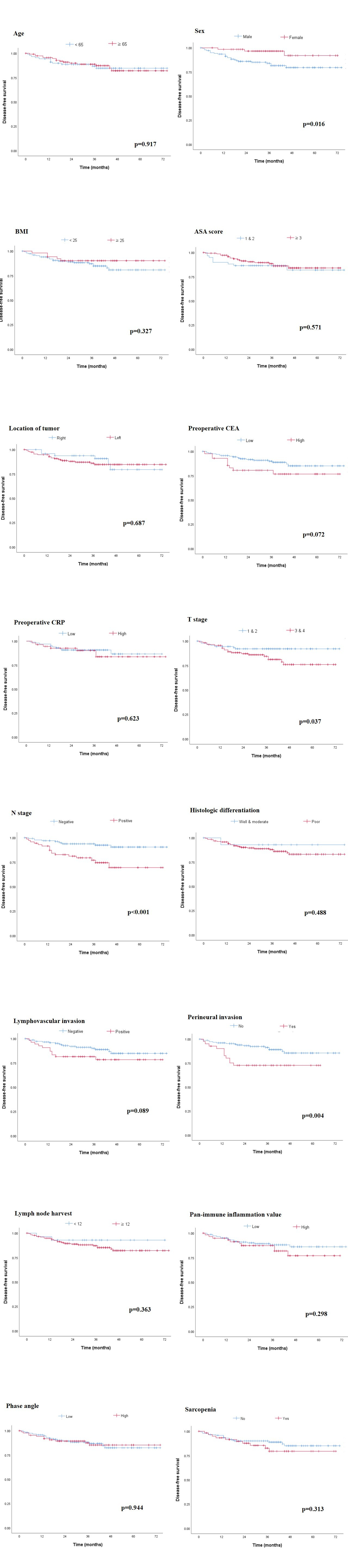

Supplement: Supplementary file 1 [file nutrients-14-03971-s001.zip › Supplementary-Figure S2.tiff]
